# LSD1 inhibitors for cancer treatment: Focus on multi-target agents and compounds in clinical trials

**DOI:** 10.3389/fphar.2023.1120911

**Published:** 2023-02-02

**Authors:** Beatrice Noce, Elisabetta Di Bello, Rossella Fioravanti, Antonello Mai

**Affiliations:** ^1^ Department of Chemistry and Technology of Drugs, Sapienza University of Rome, Rome, Italy; ^2^ Pasteur Institute, Cenci-Bolognetti Foundation, Sapienza University of Rome, Rome, Italy

**Keywords:** histone demethylases, epigenetics, LSD1 inhibitors, dual-targeting compounds, cancer therapy

## Abstract

Histone lysine-specific demethylase 1 (LSD1/KDM1A) was first identified in 2004 as an epigenetic enzyme able to demethylate specific lysine residues of histone H3, namely H3K4me1/2 and H3K9me1/2, using FAD as the cofactor. It is ubiquitously overexpressed in many types of cancers (breast, gastric, prostate, hepatocellular, and esophageal cancer, acute myeloid leukemia, and others) leading to block of differentiation and increase of proliferation, migration and invasiveness at cellular level. LSD1 inhibitors can be grouped in covalent and non-covalent agents. Each group includes some hybrid compounds, able to inhibit LSD1 in addition to other target(s) at the same time (dual or multitargeting compounds). To date, 9 LSD1 inhibitors have entered clinical trials, for hematological and/or solid cancers. Seven of them (tranylcypromine, iadademstat (ORY-1001), bomedemstat (IMG-7289), GSK-2879552, INCB059872, JBI-802, and Phenelzine) covalently bind the FAD cofactor, and two are non-covalent LSD1 inhibitors [pulrodemstat (CC-90011) and seclidemstat (SP-2577)]. Another TCP-based LSD1/MAO-B dual inhibitor, vafidemstat (ORY-2001), is in clinical trial for Alzheimer’s diseases and personality disorders. The present review summarizes the structure and functions of LSD1, its pathological implications in cancer and non-cancer diseases, and the identification of LSD1 covalent and non-covalent inhibitors with different chemical scaffolds, including those involved in clinical trials, highlighting their potential as potent and selective anticancer agents.

## 1 Introduction

### 1.1 Structure, mechanism and biological functions of LSD1

Lysine specific demethylase 1 (LSD1, KDM1A) was discovered in 2004 by the Shi’s group (Shi et al., 2004). Up to date, lysine demethylases (KDMs) according to their sequence homology and mechanism of action have been classified into two main classes: LSDs and the Jumonji-containing (JmjC) KDMs. The first enzymes belong to a superfamily of flavin adenine-dinucleotide (FAD)-dependent amine oxidases and comprise two isoforms, LSD1, the founding member, and LSD2, latterly discovered in 2009 (Ciccone et al., 2009). On the other hand, the JmjC KDMs are Fe (II)/2-oxoglutarate (2-OG)-dependent enzymes catalyzing the demethylation of mono-, di-, and trimethyl lysines, are structurally connected with nucleic acid oxygenases, and contain more than 20 members.

**FIGURE 1:**
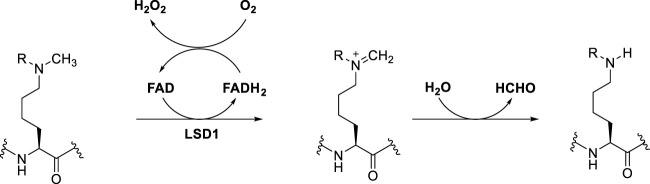
General catalytic mechanism of histone demethylation by LSD1.

LSD1 specifically removes the methyl groups from mono- and dimethyl lysine 4 or lysine 9 of histone 3 (H3K4me1/2 and H3K9me1/2), behaving as either a repressor or activator of gene expression, respectively (Rotili and Mai, 2011). Mechanistically, single-electron oxidation of the mono- or dimethylated amine at the ε-position of lysine gives an iminium cation with simultaneous reduction of FAD to FADH_2._ After FADH_2_ reoxidation to FAD by O_2_, H_2_O_2_ is generated. The iminium cation is unstable and after easy hydrolysis furnish the amine with one methyl less and formaldehyde as by-product (Dai et al., 2020) (Figure 1). The key feature that allows this mechanism to happen is the lone pair of the *N*-methylated lysine. Hence, the trimethylated species H3K4me3 is not a substrate for LSD1, since the long pair is not available.

**FIGURE 2:**
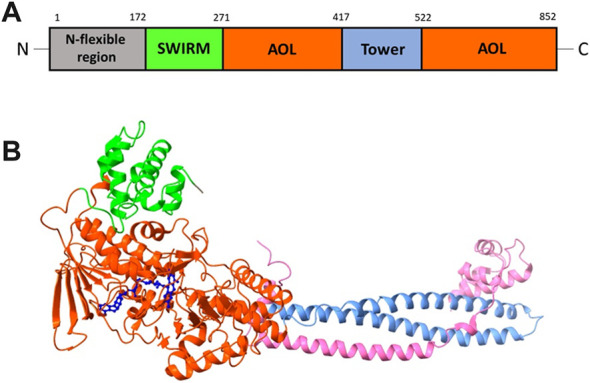
**(A)** Structural organization of LSD1 with the *N*-flexible region (grey) and the domains SWIRM (green), AOL (two parts, orange), and Tower (light blue). **(B)** X-ray structure of the LSD1/CoREST complex, with the domains SWIRM (green), AOL (orange) and Tower (light blue). The CoREST portion is depicted in pink. The FAD cofactor (blue) is located inside of the AOL domain (PDB ID: 3ABT).

LSD1 polypeptide chain can be divided into several functional regions: the *N*-flexible region; the SWIRM domain (small α-helical domain); the *C*-terminal amine oxidase domain (AOL), divided into two portions by the Tower domain (Chen et al., 2006). The *N*-flexible region has no predicted structural elements and is not necessary for catalysis, but it is essential for LSD1 nuclear localization. The SWIRM domain of LSD1 is useful as docking site to interact with other proteins and provides to retain LSD1 protein stability. It is characterized by six helical bundles and two-stranded β-sheets. These β-sheets help to form some interactions between SWIRM and AOL domains. The AOL domain - the catalytic part of LSD1 - comprises two lobes: in the first of them the oxidation reaction starts, through binding with the SWIRM domain containing the FAD-binding site, while the second one shows the site for substrate recognition. Both lobes establish a cavity in which the demethylation activity occurs into the catalytic center. The Tower domain, a typical antiparallel coiled coil, protrudes from the AOL domain and is formed by two α-helices. The Tower domain can act as an adaptor to recruit other proteins. Indeed, it is mandatory to allow the binding of the RE1-silencing transcription factor (CoREST), which is involved onto the formation of a heterodimeric complex with LSD1, increasing the enzyme activity (Figure 2) (Chen et al., 2006). This CoREST complex is important for its ability to demethylate the nucleosome complex of histones and DNA, because LSD1 alone can only demethylate the specific residue in peptides or bulk histones (Kim et al., 2020).

LSD1, when associated with CoREST, histone deacetylases (HDACs), nucleosome remodeling and deacetylation complexes, and C-terminal binding proteins, demethylates H3K4me1/2 residues and works as transcriptional co-repressor (Lee et al., 2005) (Shi et al., 2005) (Wang et al., 2009) (Shi et al., 2003). In the presence of H3K9me1/2 residues (transcription repressor markers), LSD1 acts as a transcription co-activator by demethylation, associated to androgen and estrogen receptors (Metzger et al., 2005).

### 1.2 Pathological implications of LSD1

In most human cancer cells, LSD1 plays a pivotal role in reversible cellular processes such as epithelial-mesenchymal transition (EMT) (Nieto, 2009; Ambrosio et al., 2017b), contributing to cancer invasion and progression and inducing metastatic characteristics (Choi et al., 2015). Beyond histones, the discovery of a large amount of non-histone proteins have been identified as substrates of LSD1. The tumor suppressor p53 was the first non-histone LSD1 substrate identified (Huang et al., 2007). Afterwards, it has been shown that LSD1 regulates angiogenesis, cell cycle arrest, chromatin remodeling and proliferation of cancer cells by demethylation of HIF-1α (Lee et al., 2017), E2F1 (Kontaki and Talianidis, 2010), DNMT1 (Wang et al., 2009), and STAT3 (Yang et al., 2010). Most recently, AGO2 has been reported to be demethylated by LSD1, promoting protein stability and accumulating dsRNA expression to regulate tumor T cell response (Sheng et al., 2018). Thus, LSD1 is epigenetically involved in the control of many cellular processes, such as autophagy, stemness, differentiation, senescence, cell proliferation and motility, organogenesis, (Lan et al., 2008; Zheng et al., 2015; Chen et al., 2016; Hirano and Namihira, 2016; Ambrosio et al., 2017b), haematopoiesis, and neuronal and embryonic development (Whyte et al., 2012; Karakaidos et al., 2019).

Pathologically, LSD1 is involved in metastasis and tumorigenesis and has been found dysregulated and/or overexpressed in various cancer types. LSD1 was found significantly overexpressed in many solid tumours, including prostate (Etani et al., 2019), breast (Zhou et al., 2021), small cell lung cancer (Jin et al., 2019), bladder cancer (Hayami et al., 2011), medulloblastoma (Lee et al., 2019), neuroblastoma (Ambrosio et al., 2017a), glioma (Bailey et al., 2020b), and sarcomas (Bennani-Baiti et al., 2012), as well as hematological malignancies, such as acute myeloid leukemia (AML) (Zhang et al., 2021).

For instance, in prostate cancer (PCa) LSD1 inhibition can hamper the EMT process induced by androgens, leading to a delay in the transformation of PCa into castrate-resistant prostate cancer (Wang et al., 2015). In breast cancer, LSD1 and the KDM6A UTX, a Jumonji C demethylase, are co-expressed and co-localize with estrogen receptors (ERs). Dual LSD1/UTX inhibitors exerted anticancer activity in *in vitro*, *ex vivo* and *in vivo* breast cancer models. The same compounds gave downregulation of ERα, at both transcriptional and non-transcriptional levels, through modulation of miR-181a-5p expression (Benedetti et al., 2019; Benedetti et al., 2021). The reduction of activity of LSD1, obtained by either pharmacological or genetic approach, could prevent resistance to antitumor agents in breast as well as other cancer models (Lu et al., 2018; Sobczak et al., 2022). In blood cancers, various studies demonstrated the contribution of LSD1 to the onset and progression of AML (Lokken and Zeleznik-Le, 2012). In particular, LSD1 is able to increase the leukemic stem cells (LSCs)’ clonogenic activity and start their transcriptional actions, together with a reduction of myeloid differentiation. Indeed, LSD1 inhibition increases the levels of myeloid-lineage markers, including CD11b and CD86 (Fang et al., 2017). Both *in vitro* and *in vivo* studies reported the effects of LSD1 inhibitors in terms of anti-leukemic activity, reduction of LSCs growth, induction of cell differentiation and increased survival in mouse models of AML (Chen et al., 2012).

LSD1 overexpression has been also linked to resistance to chemotherapy, immunotherapy, and radiotherapy (Dagogo-Jack and Shaw, 2018). In varicella zoster virus (VZV) and herpes simplex virus (HSV), LSD1 depletion or inhibition led to repressive chromatin and viral cycle inhibition (Gu and Roizman, 2009) (Zhou et al., 2010). In sensory neurons, LSD1 inhibitors can block the HSV reactivation from latency, highlighting LSD1 as a crucial player in viral infection and reactivation (Liang et al., 2009). Finally, LSD1 has been reported to be involved in metabolic diseases (Hino et al., 2012) and in central nervous system disorders, such as Alzheimer’s disease and depression (Rusconi et al., 2016).

### 1.3 LSD1 and immunotherapy

Resistance to immunotherapy is a complex and difficult mechanism and involves two types of cells, cancer cells and immune cells (Sharma et al., 2017). Overcoming resistance to immunotherapy means improving the ability of immune cells to recognize cancer cells, killing them (Schoenfeld and Hellmann, 2020). Targeting LSD1 could cause this effect, in particular reactivating critical immune checkpoint regulators and modulating T cells in cancer (Sheng et al., 2018) (Qin et al., 2019). Moreover, the inhibition of LSD1 suppressed stem cell-like properties and sensitized head and neck squamous cell carcinoma to PD-1 blockade (Han et al., 2021). For this reason, the combination of LSD1 inhibitors and anti-PD-1 agents could have a synergistic effect respect to the single-target inhibitor.

### 1.4 LSD1 and viral infections

LSD1, in addition to the previously citated implications, has an important role in the viral transcription. In 2001, Sakane et al. (2011) showed that the LSD1/CoREST complex is linked to the HIV promoter and triggers, through K51 demethylation, the activity of Trans-Activator of Transcription (TAT), a regulatory protein essential for HIV replication. The involvement of LSD1 in viral infections of Herpes Virus was discovered in 2010. The α-herpesviruses, including HSV and VZV, after infection use cellular transcriptional coactivator host cell factor-1 to recruit LSD1 on the immediate early promoter. LSD1 inhibition leads to the accumulation of the chromatin in its condensed state blocking the viral cycle.

### 1.5 LSD1 scaffolding functions

In addition to their inhibition of the LSD1 catalytic activity, some LSD1 inhibitors exert scaffolding functions leading, for instance, to the removal of the NK cells lytic capacity, through a potent reduction of oxidative phosphorylation (Bailey et al., 2020a), or to the disruption of the DRED complex, with increases of γ-globin and cellular HbF contents *in vitro* and *in vivo*, useful for innovative treatment of sickle cell disease (Holshouser et al., 2020).

LSD1 is overexpressed in many cancers, among which AML is one of the most represented (Schenk et al., 2012). LSD1 inhibitors gave highly variable results in AML cells, their effect can be improved by co-treatment with other drugs, including retinoic acid (RA) (McGrath et al., 2016).

It was demonstrated that the treatment with LSD1 inhibitors of cells of Acute Promyelocytic Leukemia (APL), a subtype of AML, does not induce growth arrest but increase sensitivity of the same cells to physiological concentrations of RA (Binda et al., 2010). In particular, in a panel of 21 AML cell lines (representing all subtypes), it was seen that the combination of RA with the LSD1 inhibitor MC2580 (**4**, see below) impacts on the viability of both LSD1 inhibitors-sensitive and -resistant AML cell lines. The RA treatment, joined to LSD1 inhibition, gives a differentiation not always dependent on changes in methylation levels of H3K4. The non-enzymatic role of LSD1 leads to a block of cell differentiation, but it can be removed by combination of LSD1 inhibitors with RA (Ravasio et al., 2020).

The combination between another LSD1 inhibitor (DDP38003) and RA was tested *in vivo*. All the control mice died within 3 weeks (median survival: 21 days). Treatments with RA alone or LSD1 inhibitor alone prolonged mice survival (median survival: 49 (RA) or 37 (LSD1i) days). The combination of RA and the LSD1 inhibitor strongly increased the therapeutic effect of the single agents alone (median survival, 70 days) (Ravasio et al., 2020).

## 2 LSD1 inhibitors

Since the discovery of LSD1 in 2004, many LSD1 inhibitors have been described in literature, acting through covalent or non-covalent mechanism, alone or in combination with other therapeutic agents, to fight cancer and non-cancer diseases (Hojfeldt et al., 2013; Yang et al., 2018). Some of them have undergone clinical trials for cancer treatments.

### 2.1 Covalent LSD1 inhibitors

**FIGURE 3:**
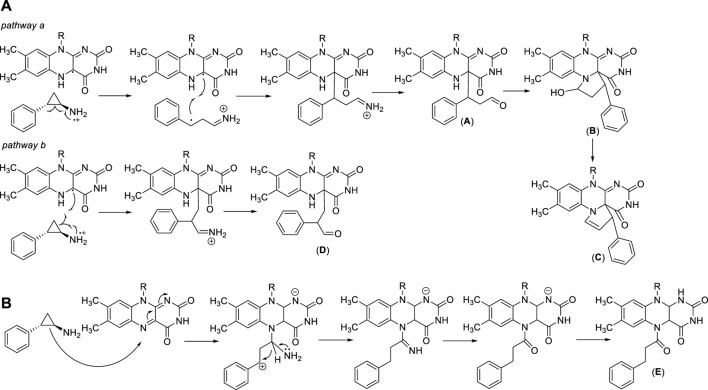
TCP-FAD adducts: proposed structures and mechanisms of formation with links at FAD C(4a) **(A)** or N(5) **(B)** position.

*Trans* 2-phenylcyclopropylamine (tranylcypromine, TCP), known with the brand name of Parnate^®^, was first approved by the US Food and Drug Administration (FDA) in 1961 for patients with major depressive disorders. It displays high inhibitory activity against MAOs (K_i_ values = 19 (MAO-A) and 16 (MAO-B) μM) (Binda et al., 2010). Since the catalytic domain of LSD1 is similar at structural level with that of MAOs, and the molecular mechanism of catalysis is common between LSD1 and MAOs, several MAO inhibitors including TCP, pargyline, and phenelzine, were tested against LSD1. Phenelzine is a non-selective, irreversible MAO inhibitor in clinical use as antidepressant and anxiolytic agent. It has been reported to inhibit also LSD1 (Culhane et al., 2010), and entered clinical trials in combination with Abraxane, a nano formulation of Paclitaxel bound to albumin, for the treatment of metastatic or advanced breast cancer (Table 1). Early experimental findings highlighted TCP as the most valuable fragment despite its moderate LSD1 inhibitory activity (K_i_ = 271 µM) (Binda et al., 2010). Since TCP is an already approved drug, its repurposing as anticancer agent is a valuable strategy for drug discovery (Song et al., 2022). It has been postulated that TCP inactivates LSD1 through a single-electron transfer (SET) mechanism (Figure 3A). Depending on the cyclopropyl C-C bond involved in the homolysis, four different TCP-FAD adducts can be obtained through two distinct pathways. One initial step may consist in the opening of the cyclopropyl C1-C2 bond with the transfer of a single electron from the primary amine nitrogen of TCP to FAD, allowing the formation of a radical cation, followed by the formation of a stabilized benzyl radical. Such radical interacts with the C(4a) of the FAD cofactor leading to an iminium ion yielding the 3-phenylpropionaldehyde (**A**) after hydrolysis. Hence, this aldehyde intramolecularly can react with the N(5) of FAD to afford a hemiaminal intermediate (**B**), which gives the final unsaturated cyclic adduct (**C**) after dehydration (Figure 3A, *pathway a*). The second pathway involves the opening of the cyclopropyl C1-C3 bond, with the formation of a radical carbon and a concerted bond formation with the FAD C(4a) position leading to the production of a 2-phenylpropyl iminium ion, subsequently hydrolyzed to the corresponding aldehyde (**D**) (Figure 3A, *pathway b*). Despite the second route is energetically unfavorable, the adduct (**D**) is the major product detected and isolated during the MAO-B covalent inhibition by TCP (Schmidt and McCafferty, 2007; Yang et al., 2007). In 2010, we solved the crystal structures of (−)-TCP, (+)-TCP, (−)-4-Br-TCP, (+)-4-Br-*cis* 2-phenylcyclopropylamine and the two more potent LSD1 inhibitors **4** and **5** (see below) in complex with LSD1/CoREST (Binda et al., 2010), and we observed the formation of adducts in which the bond with the FAD factor occurred at the N(5) instead of the C(4) position, as proposed by Mimasu et al., in 2008 (Mimasu et al., 2008). The mechanism postulated for the formation of the N(5) adducts involves the attach of the C1-C2 bond of TCP on the N(5) position of FAD, with an initial formation of a stable benzylic cation. After, a 1,3 hydride shift occurs followed by hydrolysis of the iminium cation giving the adduct (**E**) in which FAD is acylated at N(5) (Figure 3B).

**TABLE 1:** Overview of LSD1 inhibitors currently in clinical investigations for cancer treatment.

Compound	Organization	Study title	Indication	Status
Tranylcypromine (TCP)	University of Miami; Women’s Cancer Association; Gabrielle’s Angel Foundation	Study of TCP-ATRA for Adult Patients With AML and MDS (TCP-ATRA)	Acute Myelogenous Leukemia, Myelodysplastic Syndromes Leukemia	Phase 1, Completed NCT02273102
	Universitaetsklinikum Halle, Halle, Germany	Phase I/II Trial of ATRA and TCP in Patients with Relapsed or Refractory AML and no Intensive Treatment is Possible	Acute Myeloid Leukemia (TCP-AML)	Phase 1/2, Recruiting NCT02261779
	Universitätsklinikum Heidelberg; Universitätsklinik Düsseldorf, Medical School Duesseldorf; Universitätsklinikum Frankfurt Main, Medical School Frankfurt; Universitätsklinikum Freiburg, Medical School Freiburg; Klinikum München rechts der Isar, Medical School Munich rechts der Isar; Universitätsklinikum Tübingen, Medical School Tuebingen	Study of Sensitization of Non-M3 AML Blasts to ATRA by Epigenetic Treatment With Tranylcypromine (TCP) (TRANSATRA)	Acute Myeloid Leukemia Myelodysplastic Syndrome	Phase 1/2, Recruiting NCT02717884
ORY-1001 Iadademstat	Fox Chase Cancer Center; Oryzon Genomics S.A.	Iadademstat in Combination with Paclitaxel in Relapsed/Refractory SCLC and Extrapulmonary High Grade NET	Small-cell Lung Cancer, Neuroendocrine Carcinoma	Phase 2, Not yet recruiting NCT05420636
	Oryzon Genomics S.A.	Study of Iadademstat and Gilteritinib in Patients With R/R AML With FMS-like Tyrosine Kinase Mutation (FLT3 Mut+) (FRIDA)	Acute Myeloid Leukemia, in Relapse Acute Myeloid Leukemia Refractory	Phase 1, Not yet recruiting NCT05546580
Bomedemstat (IMG-7289)	Imago BioSciences, Inc.	IMG-7289 in Patients with Essential Thrombocythemia	Essential thrombocythemia	Phase 2, Active, not recruiting NCT04254978
	Terrence J Bradley, MD; Imago BioSciences, Inc.	IMG-7289 in Patients with Essential Thrombocythemia (ET) or Polycythemia Vera (PV)	Essential thrombocythemia, Polycythemia Vera	Phase 2, Recruiting NCT04262141
	Imago BioSciences,Inc.	IMG-7289 in Patients with Myelofibrosis	Myelofibrosis, Post-polycythemia Vera Myelofibrosis (PPV-MF), Post-essential Thrombocythemia Myelofibrosis (PET-MF), Primary Myelofibrosis (PMF)	Phase 2, Completed NCT03136185
	The University of Texas Health Science Center at San Antonio; Imago BioSciences,Inc.	Hematology, IMG-7289, LSD1 (Lysine-Specific Demethylase 1) Inhibitor, Essential Thrombocythemia (ET), Ph 2	Thrombocythemia, Essential	Phase 2, Recruiting NCT04081220
	Imago BioSciences, Inc.	IMG-7289, with and without ATRA, in Patients with Advanced Myeloid Malignancies	Acute Myeloid Leukemia, Myelodysplastic Syndrome	Phase 1, Completed NCT02842827
	Imago BioSciences, Inc.	Extension Study of Bomedemstat (IMG-7289) in Patients with Myeloproliferative Neoplasms	Thrombocythemia, Essential Primary Myelofibrosis	Phase 2, Recruiting NCT05223920
	University of Washington; National Cancer Institute (NCI); Imago BioSciences, Inc.	Bomedemstat and Maintenance Immunotherapy for Treatment of Newly Diagnosed Extensive Stage Small Cell Lung Cancer	Extensive Stage Lung Small Cell Carcinoma, Limited Stage Lung Small Cell Carcinoma	Phase 1/2, Recruiting NCT05191797
	Imago BioSciences, Inc.	Bomedemstat in Patients with Polycythemia Vera	Polycythemia Vera	Phase 2, Not yet recruiting NCT05558696
	The University of Hong Kong; Imago BioSciences, Inc.	Bomedemstat (IMG-7289) Plus Ruxolitinib for Myelofibrosis	Myelofibrosis	Phase 2, Recruiting NCT05569538
	Terrence J Bradley, MD; Imago BioSciences, Inc.	Venetoclax and Bomedemstat in Patients with Relapsed/Refractory Acute Myeloid Leukemia (VenBom)	Acute Myeloid Leukemia Refractory Acute Myeloid Leukemia Acute Myeloid Leukemia, in Relapse	Phase 1, Recruiting NCT05597306
GSK-2879552	GlaxoSmithKline; Parexel	Safety, Clinical Activity, Pharmacokinetics (PK) and Pharmacodynamics Study of GSK2879552, Alone or with Azacitidine, in Subjects with High Risk Myelodysplastic Syndromes (MDS)	Myelodysplastic Syndromes	Phase 1/2, Terminated (The risk benefit in the study population does not favour continuation of the study) NCT02929498
	GlaxoSmithKline	Investigation of GSK2879552 in Subjects with Relapsed/Refractory Small Cell Lung Carcinoma	Carcinoma, Small Cell	Phase 1, Terminated (The risk benefit in relapsed refractory SCLC does not favour continuation of the study) NCT02034123
	GlaxoSmithKline	A Phase I Dose Escalation Study of GSK2879552 in Subjects with Acute Myeloid Leukemia (AML)	Leukaemia, Myelocytic, Acute	Phase 1, Terminated (The risk benefit in relapsed refractory AML does not favour continuation of the study) NCT02177812
INCB-059872	Incyte Corporation	A Study to Evaluate Safety, Pharmacokinetic, and Biological Activity of INCB059872 in Subjects with Sickle Cell Disease	Sickle Cell Disease	Phase 1, Terminated (This study is terminated due to a business decision not to pursue INCB059782 in Sickle Cell Disease indication) NCT03132324
		A Study of INCB059872 in Relapsed or Refractory Ewing Sarcoma	Relapsed Ewing Sarcoma	Phase 1, Terminated (Strategic Business Decision) NCT03514407
		An Open-Label, Dose-Escalation/Dose-Expansion Safety Study of INCB059872 in Subjects with Advanced Malignancies	Solid Tumors and Hematologic Malignancy	Phase 1/2, Terminated (Strategic Business Decision) NCT02712905
		Azacitidine Combined with Pembrolizumab and Epacadostat in Subjects with Advanced Solid Tumors (ECHO-206)	Solid Tumors, Advanced Malignancies, Metastatic Cancer	Phase 1/2, Terminated (Study terminated by Sponsor) NCT02959437
Pulrodemstat (CC-90011)	Celgene	A Study of CC-90011 and Comparators in Participants with Prostate Cancer	Prostatic Neoplasms	Phase 1, Recruiting NCT04628988
		A Safety and Efficacy Study of CC-90011 in Combination with Nivolumab in Subjects with Advanced Cancers	Neoplasms	Phase 2, Active, not recruiting NCT04350463
		A Safety, Tolerability and Preliminary Efficacy Evaluation of CC-90011 Given in Combination with Cisplatin and Etoposide in Subjects with First Line, Extensive Stage Small Cell Lung Cancer	Small Cell Lung Carcinoma	Phase 1, Active, not recruiting NCT03850067
		A Safety, Tolerability and Preliminary Efficacy Study of CC-90011 in Combination with Venetoclax and Azacitidine in R/R Acute Myeloid Leukemia and Treatment-naïve Participants Not Eligible for Intensive Therapy	Leukemia, Myeloid	Phase 1, Completed NCT04748848
		A Safety and Efficacy Study of CC-90011 in Participants with Relapsed and/or Refractory Solid Tumors and Non-Hodgkin’s Lymphomas	Lymphoma, Non-Hodgkin Neoplasms	Phase 1, Recruiting NCT02875223
Seclidemstat (SP-2577)	Salarius Pharmaceuticals, LLC	Phase 1 Trial of the LSD1 Inhibitor SP-2577 (Seclidemstat) in Patients with Advanced Solid Tumors	Advanced Solid Tumors	Phase 1, Completed NCT03895684
	Salarius Pharmaceuticals, LLC	A Rollover Protocol to Allow for Continued Access to the LSD1 Inhibitor Seclidemstat (SP-2577)	Ewing Sarcoma, Myxoid Liposarcoma, Desmoplastic Small Round Cell Tumor, Extraskeletal Myxoid Chondrosarcoma, Angiomatoid Fibrous Histiocytoma, Clear Cell Sarcoma, Myoepithelial Tumor, Low Grade Fibromyxoid Sarcoma, Sclerosing Epithelioid Fibrosarcoma	Phase 1/2, Enrolling by invitation NCT05266196
	Salarius Pharmaceuticals, LLC; National Pediatric Cancer Foundation	Clinical Trial of SP-2577 (Seclidemstat) in Patients with Relapsed or Refractory Ewing or Ewing-related Sarcomas	Ewing Sarcoma, Myxoid Liposarcoma, Sarcoma, Soft Tissue, Desmoplastic Small Round Cell Tumor, Extraskeletal Myxoid Chondrosarcoma, Angiomatoid Fibrous Histiocytoma, Clear Cell Sarcoma, Primary Pulmonary Myxoid Sarcoma, Myoepithelial Tumor, Sclerosing Epithelioid Fibrosarcoma, Fibromyxoid Tumor	Phase 1, Recruiting NCT03600649
	M.D. Anderson Cancer Center	Seclidemstat and Azacitidine for the Treatment of Myelodysplastic Syndrome or Chronic Myelomonocytic Leukemia	Chronic Myelomonocytic Leukemia-0, Chronic Myelomonocytic Leukemia-1, Chronic Myelomonocytic Leukemia-2, Myelodysplastic Syndrome, Recurrent Chronic Myelomonocytic Leukemia, Recurrent Myelodysplastic Syndrome	Phase 1/2, Recruiting NCT04734990
	HonorHealth Research Institute - Merck Sharp & Dohme LLC - Salarius Pharmaceuticals, LLC	Pilot Trial of SP-2577 Plus Pembrolizumab in Select Gynecologic Cancers	SCCOHT, Ovarian Clear Cell Tumor, Ovarian Endometrioid Adenocarcinoma, Endometrial Cancer	Phase 1, Withdrawn NCT04611139
JBI-802	Jubilant Therapeutics Inc.	A Study of Orally Administered JBI-802, an LSD1/HDAC6 Inhibitor, in Patients with Advanced Solid Tumors	Locally Advanced Solid Tumor, Metastatic Solid Tumor	Phase 1/2, Recruiting NCT05268666
Phenelzine	EpiAxis Therapeutics; The Canberra Hospital; Southern Medical Day Care Centre; Liverpool Cancer Therapy Centre	An Early Phase Study of Abraxane Combined with Phenelzine Sulfate in Patients with Metastatic or Advanced Breast Cancer (Epi-PRIMED)	Metastatic Breast Cancer	Phase 1, Completed NCT03505528

The covalent TCP-FAD adduct in LSD1 is in a hydrophobic pocket surrounded by Val333, His564, Thr335, Tyr761, Ala809, and Thr810 residues. The benzene ring of the adduct forms weak Van Der Waals bonds with the methyl groups of Thr810 and Thr335, but does not have other interactions with the previously named hydrophobic residues (Yang et al., 2007). For this reason, extensive structure-activity relationship (SAR) studies led to the discovery of numerous TCP-based irreversible LSD1 inhibitors with increased potency and selectivity, able to form strong covalent adducts with the flavin cofactor (Lee et al., 2006; Schmidt and McCafferty, 2007; Zheng et al., 2016).

We will summarize the results obtained with such TCP-based LSD1 inhibitors, and discuss their potential use as therapeutic drugs, with a focus on multi-targeting inhibitors and on compounds in clinical trials. We grouped these compounds into two clusters: i) derivatives substituted on the TCP phenyl ring, and ii) derivatives substituted on the TCP primary amino group.

#### 2.1.1 Compounds substituted on the TCP phenyl ring

**FIGURE 4:**
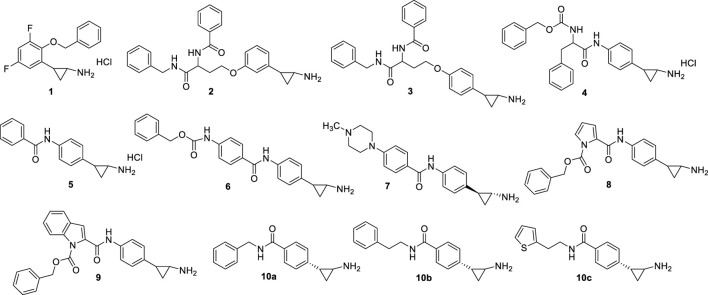
Covalent, TCP-based LSD1 inhibitors substituted on the phenyl ring.

From 2009, the TCP benzene ring has been substituted at *ortho*, *meta*, and/or *para* positions to produce potent LSD1 inhibitors. In 2010, Mimasu *et al.* described some derivatives with (cyclo)alkyl/arylalkyloxy residues at the *ortho* and eventually *para* positions, and halogen atoms at the *meta* position(s), of the TCP phenyl ring (Mimasu et al., 2010). The best result was obtained with **S2101** (**1**) (Figure 4), an *ortho*-benzyloxy-*meta*/*meta*-difluoro substituted derivative exhibiting stronger anti-LSD1 potency than TCP (K_i_ = 0.61 μM). In 2009 and 2010, Ueda et al. (2009) and Binda et al. (2010) reported the same strategy to obtain potent LSD1 inhibitors: the insertion at *para* (Ueda, Binda) or *meta* (Ueda) positions of the TCP phenyl ring of hindered and branched amino acid-based substituents, linked through an ether (Ueda, **NCL1** (**2**), **NCL2** (**3**), Figure 4) or amide (Binda, **MC2580** (**4**), Figure 4) function. NCL compounds revealed to be potent LSD1 inhibitors selective over MAOs (IC_50_ values: NCL1 = 2.5 (LSD1), 230 (MAO-A), 500 (MAO-B) µM; NCL2 = 1.9 (LSD1), 290 (MAO-A), >1,000 (MAO-B) µM), due to the presence of large groups attached to the TPC phenyl ring which cannot be accommodated by the MAOs active sites. The same compounds showed interesting anticancer properties, with a range of reduction of 50% cell growth from 6 to 67 μM in a panel of cancer cell lines (HeLa cervical cancer, HCT-116 colon cancer, PC-3 prostate cancer, KYSE-150 esophageal squamous cell carcinoma, and SH-SY5Y neuroblastoma). In Binda *et al.*, the *para* position of the TCP ring was substituted with a simple aroyl amine or a *N*-benzyloxycarbonylamino acyl amine. **MC2584** (**5**) and MC2580 (**4**) were identified as first lead compounds, with a K_i_ of 1.1 and 1.3 μM, respectively, and selectivity over MAO-B for MC2580. In APL cells, MC2580 displayed antiproliferative activity in synergy with RA (Binda et al., 2010). These two prototypes were after developed to furnish more potent, cell permeable and selective inhibitors (Fioravanti et al., 2020). Among them, **MC3340** (**6**) (Figure 4) exhibited IC_50_^LSD1^ = 90 nM with selectivity over MAOs, and submicromolar potency against APL NB4 (IC_50_ = 0.6 μM) and AML MV4-11 (IC_50_ = 0.4 μM) cells (Fioravanti et al., 2020), and the *1S*, *2R* pure enantiomer **7** (Vianello et al., 2016) (Figure 4) showed IC_50_^LSD1^ = 84 nM, high selectivity over MAO-B, and huge anticlonogenic activity in mouse APL blasts and THP-1 cells. This last compound, when orally administered *in vivo* in a mouse APL model, appreciably increased survival of the treated mice (35% and 62% at the doses of 11.25 and 22.50 mg/kg, respectively) without apparent toxicity (Vianello et al., 2016). The replacement of the TCP benzene ring with pyrrole and indole moieties afforded **MC3288** (**8**) and **MC3382** (**9**) (Figure 4), which showed significant LSD1 inhibitory activity (IC_50_ = 0.032 µM and IC_50_ = 0.040 µM, respectively) and antiproliferative effects at low micromolar level against AML and APL cells (Rodriguez et al., 2015).

In 2021, novel TCP analogues with a carboxamide at the *para* position of the benzene ring were developed by Borrello *et al.*. Some of them, containing a benzyl-, 2-phenethyl-, or 2-thienylethylamino carboxamide portion (**10a-c**) (Figure 4) were submicromolar inhibitors of the enzyme (10a: IC_50_^**10a**^ = 0.3 µM; IC_50_^**10b**^ = 0.4 µM; IC_50_^**10c**^ = 0.6 µM) (Teresa Borrello et al., 2021).

##### 2.1.1.1 Multi-target LSD1 inhibitors

**FIGURE 5:**
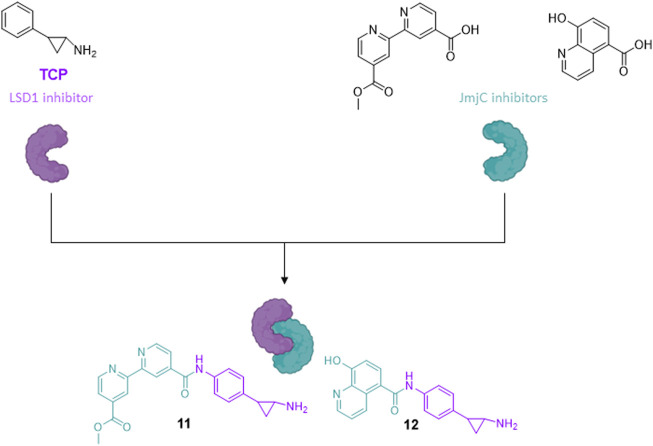
Hybrid compounds inhibitors of LSD1 and JmjC.

Very often the epigenetic targets work in macromolecular and multicomponent complexes, formed by recruitment of different proteins and/or factors, able to activate or silence transcription. The idea to simultaneously inhibit different components of repressive complexes through a unique hybrid molecule could lead to a synergistic action for cancer treatment, by disruption of the complex and reactivation of transcription of tumor-suppressor genes. As in prostate cancer the two families of KDMs, LSD1 and JmjC enzymes (specifically, KDM4A/C), are co-expressed and colocalized with the androgen receptor, the design and synthesis of pan-demethylase inhibitors, able to hit at the same time both the families of demethylases, has a strong rationale for treatment of prostate cancer. Thus, the LSD1-inhibiting TCP portion was coupled with fragments able to inhibit the JmjC enzymes, such as the 4-carboxy-4′-carbomethoxy-2,2′-bipyridine and the 5-carboxy-8-hydroxyquinoline, to obtain multitargeting inhibitors (**11** and **12**) (Rotili et al., 2014). The two hybrid compounds induced arrest of proliferation and huge dose-dependent apoptosis in prostate cancer LNCaP and colon cancer HCT-116 cell lines, with slight or no apoptosis induction in mesenchymal progenitor cells, suggesting a cancer selectivity for these compounds. In the same cancer cell lines, **11** and **12** showed increased H3K4 and H3K9 methylation levels, confirming the inhibition of the two targets at the cellular level (Figure 5) (Rotili et al., 2014).

LSD1 physically interacts with the co-repressor CoREST and with HDAC1/2 to form a repressive CoREST complex (Pilotto et al., 2015; Zheng et al., 2015). Thus, the simultaneous blockage of LSD1 and HDAC1/2 represents a promising strategy to reactivate transcription against cancer, leading to combination studies and hybrid compounds development. The combination of TCP with the zinc-binding group of Entinostat, a well-known HDAC inhibitor belonging to the benzamide series and inhibiting specifically HDAC1-3, furnished hybrid molecules targeting the ternary CoREST complex and inhibiting both LSD1 and HDAC1 at submicromolar doses (Kalin et al., 2018).

**FIGURE 6:**
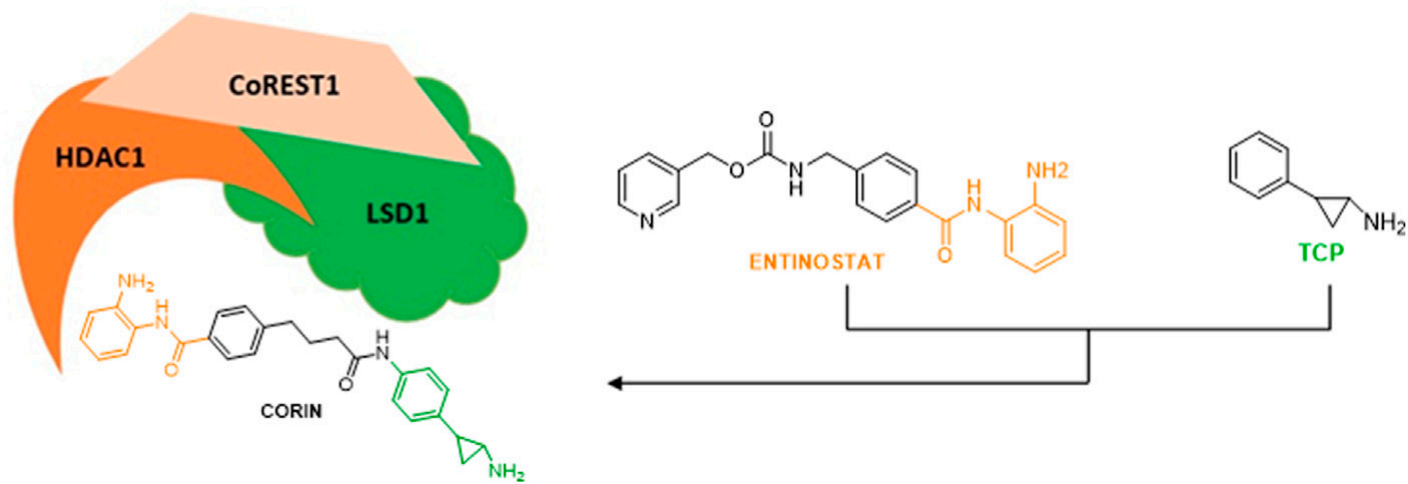
Schematic figure of CoREST1 complex and the inhibitor Corin.

In a panel of melanoma cells, the dual LSD1/HDAC compound **Corin** (Figure 6) (Kalin et al., 2018) exhibited higher inhibition of proliferation with respect to the relative single-target inhibitors (TCP for LSD1 and Entinostat for HDACs) and to their combination. Corin showed good pharmacokinetics and safety profile, and in melanoma SK-MEL-5 BALB/c mice it reduced by 61% tumor growth after 28 days without relevant toxicity. Moreover, Corin increased H3K9 acetylation and H3K4 dimethylation, induced some genes related to anticancer effects such as p21, CHOP, and MXD1, and reduced the level of the marker of proliferation Ki67 (Kalin et al., 2018). In diffuse intrinsic pontine glioma (DIPG) Corin was equally effective *in vitro* and *in vivo*, demonstrating the crucial effect of dual LSD1/HDAC inhibition also in this context (Anastas et al., 2019).

#### 2.1.2 Compounds substituted on the TCP amino group

**FIGURE 7:**
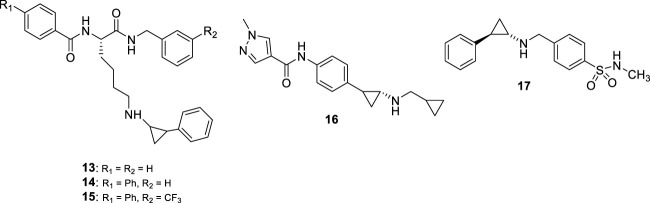
Covalent TCP-based LSD1 inhibitors substituted on the amino group.

Most of the LSD1 inhibitors currently undergoing clinical studies are TCP derivatives alkylated on the nitrogen atom. In 2013, some authors reported cyclic or linear peptides as LSD1 inhibitors (Tortorici et al., 2013; Kumarasinghe and Woster, 2014). Among them, Ogasawara *et al.* starting from the assumption that a peptide chain could be the vector of TCP gaining LSD1 inhibition, synthesized a series of peptide derivatives, including TCP-Lys-4 H3-21 showing potent LSD1 inhibition (IC_50_ = 0.16 μM) and >625 selectivity index over MAOs (Ogasawara et al., 2013). However, it was not very effective on cancer cells (GI_50_ values: 27 μM (HeLa) and >160 μM (SH-SY5Y)), probably due to poor cell permeability (Ogasawara et al., 2013). Afterwards, the same research group showed that the length of the peptide chain was very important for the inhibitory potency of compounds (Kakizawa et al., 2015). Thus, they reduced the peptide itself to a unique lysine residue, with acyl and amino groups at the α-amino and carboxy functions, and with the insertion of a phenylcyclopropyl moiety at the ε-amino group. The obtained NCD compounds exhibited submicromolar inhibitory activity against LSD1 (IC_50_ values: 0.30 (**NCD18** (**13**)), 0.48 (**NCD25** (**14**)) and 0.58 (**NCD41** (**15**)) µM) (Figure 7), with preferences for either the 1*R*, 2*S* (NCD18 and NCD25) or 1*S*, 2*R* (NCD41) enantiomer, reflecting a different preferred binding mode in the LSD1 catalytic pocket. Among the three compounds, NCD41 displayed the highest antiproliferative activity against HeLa cervical cancer (GI_50_ = 4.1 μM) and neuroblastoma SH-SY5Y (GI_50_ = 2.4 μM) cells (Itoh et al., 2014).

In 2017, Takeda Pharmaceuticals disclosed a novel LSD1 inhibitor, **T-3775440** (**16**) (Figure 7) showing potent LSD1 inhibition (IC_50_ = 20 nM) (Ishikawa et al., 2017a). In experimental studies, T-3775440 highly induced growth arrest in Acute Megakaryoblastic Leukaemia and AML cells through LSD1 inhibition. Moreover, the co-treatment of T-3775440 with the NEDD8-activating enzyme inhibitor pevonedistat displayed synergistic inhibition against AML cells (Ishikawa et al., 2017b).

In 2018, a number of *N*-alkylated TCP derivatives were synthesized, and some of them were found to be very potent (Schulz-Fincke et al., 2018). Among them, the *N*-methyl sulfonamide **17** (Figure 7) was the most potent LSD1 inhibitor (IC_50_ = 0.19 μM), with 90-fold selectivity over MAO-A and no inhibition towards MAO-B. Furthermore, such derivative was able to inhibit the formation of colonies of leukemic cells. Docking studies revealed that the large LSD1 binding pocket can admit the selective binding of lipophilic as well as polar groups, whereas in MAO-A the binding site is tighter and characterized by many lipophilic residues that do not allow the stacking with polar substituents (Schulz-Fincke et al., 2018). Other important TCP-based compounds substituted at the primary amino group entered clinical trials for the treatment of cancer diseases (see below).

#### 2.1.3 TCP-based inhibitors in clinical trials

The co-treatment of TCP and all-*trans*-retinoic acid (ATRA) is present in three clinical trials against AML and myelodysplastic syndromes (MDS) (Table 1). The combination therapy of TCP and ATRA displayed increased antileukemic effect compared with that of each drug alone (Wass et al., 2021).

**FIGURE 8:**
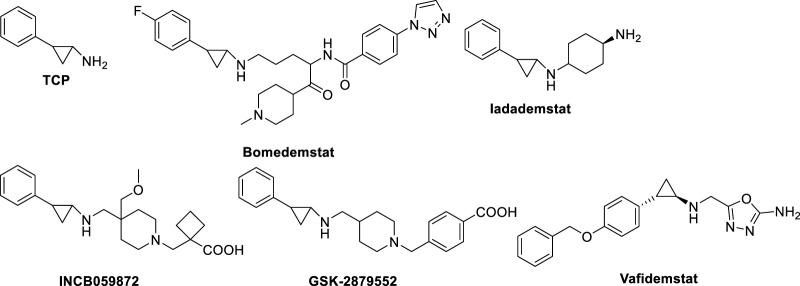
Irreversible LSD1 inhibitors in clinical trials for cancer treatment.

**ORY-1001 (Iadademstat**, RG6016, R07051790) (Figure 8) is a potent LSD1 inhibitor (IC_50_ = 18 nM) developed by Oryzon Genomics, acting through irreversible binding to the LSD1 FAD cofactor. ORY-1001 is currently in clinical trials (Table 1) for the treatment of AML and solid tumors (Salamero et al., 2020). It is highly selective for LSD1 over MAOs and displayed unrivaled sub-nanomolar cellular activity in differentiation assays. In particular, it gave time and dose-dependent induction of the CD11b differentiation marker in mixed lineage leukemia (MLL)-AF9 cells. Interestingly, such effect preceded changes in H3K4me2 levels. Moreover, ORY-1001 decreased colony formation particularly on MLL-translocated cells, but also on other AML cells. In melanoma, the combination of ORY-1001 with an anti-PD1 antibody used for 22 days led to significant tumor growth inhibition, 54% higher than that with group treated with the anti-PD1 antibody alone (Maes et al., 2019).

**ORY-2001,** or **Vafidemstat** (Figure 8), was reported by Oryzon Genomics and it is an orally bioavailable dual inhibitor of LSD1 and MAO-B (Cavalcanti et al., 2022). Due to this dual effect and its capability to cross the blood-brain barrier, ORY-2001 entered in 2019 the phase IIa clinical trial for the treatment of mild to moderate Alzheimer’s diseases (Mehndiratta and Liou, 2020), it being the only LSD1 inhibitor with an indication for treatment of neurogenerative diseases. In preclinical and clinical studies, ORY-2001 has been shown to be able to reestablish behavior impairments, decrease aggressiveness and social withdrawal and gain memory (EudraCT 2018-002140-88; EudraCT 2019-001436-54). In addition, ORY-2001 was effective in treatment of multiple sclerosis (EudraCT 2017-002838-23) and it represents the first epigenetic approach against this disease. Moreover, since in COVID-19 many inflammatory cytokines with immunomodulatory action are central to trigger the cytokine storm and the following high inflammation status, it was determined the tolerability and efficacy of ORY-2001 to prevent acute respiratory distress syndrome in patients with severe SARS-CoV-2 infection (EudraCT 2020-001618-39), due to the ability of ORY-2001 to reduce the levels of IL-6, IL-1β, and other relevant inflammatory cytokines. At the moment ORY-2001 is in Phase 2 clinical trials for mild to moderate Alzheimer’s Disease (NCT03867253) and for borderline personality disorder (NCT04932291).

**Bomedemstat**, also known as **IMG-7289** (Figure 8) (Hiatt et al., 2022), is an oral small molecule irreversible LSD1 inhibitor (IC_50_ = 56.8 nM) developed by Imago BioSciences, in clinical trials for myeloid-related malignancies (Table 1). It is well tolerated in heterogeneous patients with advanced myelofibrosis (MF), improves symptomatology, reduces spleen sizes, and normalizes or improves blood cell counts in some patients (Dai et al., 2020). An ongoing, multi-center, open-label study involving IMG-7289 recently moved from a Phase I/IIa dose-range finding study to a Phase IIb study of administered orally once-daily in adult patients with intermediate-2 or high-risk MF resistant or intolerant to ruxolitinib, a selective JAK inhibitor. The high doses used for Phase II study (40–60 mg/day) are about 500-fold higher than those of ORY-1001, and surpass the 10 mg/day threshold typically used in the risk assessment protocol for idiosyncratic toxicity of covalent ligands, with increased risk for off-target effects (Lammert et al., 2008; Baillie, 2021).

**GSK2879552** (Figure 8), an orally active *N*-alkylated derivate of TCP, was identified after the screening of 2.5 million compounds with an IC_50_ of 24 nM (Mohammad et al., 2015; Bauer et al., 2019), and the evaluation of its anticancer activity using more than 165 cell lines showed significant inhibition of AML and SCLC cell lines proliferation. In *in vivo* xenograft models, GSK2879552 was effective to inhibit the growth of NCI-H1417 SCLC cells without inducing thrombocytopenia and other hematologic toxicities (Mohammad et al., 2015). Two clinical phase 1 trials investigating the pharmacodynamics, pharmacokinetics, safety, and clinical activity of GSK2879552 in patients with relapsed/refractory SCLC (NCT02034123) and AML (NCT02177812) have been terminated. Besides, a phase I/II, open-label study evaluating the safety and clinical activity of GSK2879552 alone, or in combination with azacytidine, a well-known DNA methyltransferase inhibitor, in subjects with myelodysplasia, has also been terminated (NCT02929498). As shown on the website of clinicaltrials.gov, the risk/benefit ratio does not support further studies with this compound (Table 1). Additionally, GSK2879552 displayed synergy with ATRA in various subtypes of AML for cell differentiation, proliferation, and cytotoxicity (Smitheman et al., 2019).

**INCB059872** (Figure 8), developed by Imago BioSciences, is another *N*-alkylated TCP-based LSD1 inhibitor currently under clinical trials to treat various cancers (Johnston et al., 2020). Currently, there are four clinical phase 1,2 studies for cancer therapy, for sickle cell disease (NCT03132324), relapsed Ewing sarcoma (NCT03514407), solid tumors and hematologic malignancy (NCT02712905), metastatic cancer (NCT02959437). However, all these studies have been terminated for strategic business decisions (Table 1). In human SCLC preclinical models, INCB059872 was effective in inducing growth arrest at nanomolar level (EC_50_ values = 47–377 nM).

Very recently, **JBI-802** has been identified by Jubilant Therapeutics Inc. as a dual LSD1/HDAC6/8 inhibitor, featuring nanomolar inhibition of the above three targets (IC_50_ values: 50 nM (LSD1), 11 nM (HDAC6), and 98 nM (HDAC8)) and about 100-fold selectivity against other HDAC isoforms (Sivanandhan et al., 2020). In hematological cancers (such as AML, chronic lymphocytic leukemia, acute megakaryocytic leukemia, Z-138 lymphoblast cells, multiple myeloma cells) as well as in solid tumors (small cell lung cancer (SCLC) and sarcoma cells) JBI-802 displayed huge antiproliferative effect. Functional tests for target engagement studies performed both *in vitro* and *in vivo* showed significant dose-dependent increase in CD11b, CD86 and GFI1b (markers of LSD1 inhibition) and tubulin acetylation (marker of HDAC6 inhibition) levels (Sivanandhan et al., 2020). JBI-802 entered Phase 1/2 clinical trial for treatment of advanced and metastatic solid tumors (Table 1).

Additionally, **Phenelzine** (as written before) entered Phase 1 clinical trial for treatment of metastatic breast cancer in combination with nanoparticle albumin-bound Paclitaxel (Abraxane) (Table 1).

### 2.2 Reversible LSD1 inhibitors

**FIGURE 9:**
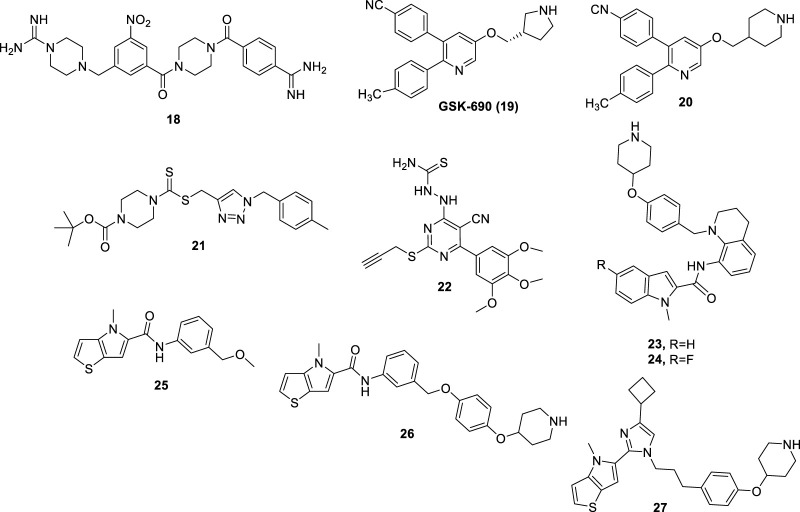
Reversible LSD1 inhibitors.

Compounds derived from TCP, showing irreversible LSD1 inhibition due to the formation of covalent bonds within the enzyme active site, are very effective but suffer from several side effects. Indeed, they can show significant affinities with several other targets beyond LSD1, including neurotransmitters, metabolizing enzymes, receptors, and transporters. Moreover, in addition to long-lasting on-target effects, they could in principle also induce prolonged off-target effects (Dai et al., 2020). Thus, researchers have turned to the design and synthesis of compounds that could establish a non-covalent bond with LSD1 and consequently should show a better safety profile. Many compounds have been designed, classified according to their chemical structure. Compound **CBB1003** (**18**) (Figure 9) (IC_50_ = 10.5 µM) is part of a group of reversible LSD1 inhibitors containing guanidinium/amidinium groups. Such positively charged functions form strong hydrogen bonds with the negatively charged amino acid residues present in the active site of LSD1, and the hydrophobic substituents dock into the deep pocket close to the FAD cofactor. In addition, the nitro group of CBB1003 is likely to form a hydrogen bonding interaction with His564 (Wang et al., 2011). In further studies, CBB1003 displayed weak cell growth inhibition (IC_50_ = 250 μM) in colorectal cancer (CRC) through downregulation of leucine-rich repeat-containing G-protein-coupled receptor 5 (LGR5), a CRC stem cell marker involved in carcinogenesis (Hsu et al., 2015).

Continuing the search for derivatives that could show a potent and reversible inhibitory action against LSD1, numerous compounds with different chemical structures have been tested. **GSK-690** (**19**) (Figure 9), a 2,3-diphenylpyridyn-5-ol derivative, was a potent LSD1 inhibitor (IC_50_ = 90 nM) *in vitro* and highly selective over MAO-A (IC_50_ >200 μM) (Hitchin et al., 2013). GSK-690, when tested in leukemia THP-1 and MV4-11 cells at 10 μM inhibited the relative clonogenic activities of almost 70% and 80%, respectively. Moreover, it demonstrated its LSD1 inhibition in cells by increasing the expression of CD86, a gene modulated by LSD1, in THP-1 cells (Mould et al., 2017).

Starting from the GSK-690 structure, numerous derivatives have been synthesized as novel LSD1 inhibitors (Wu et al., 2016). In 2015 the 3-(4-isocyanophenyl)-5-(piperidin-4-ylmethoxy)-2-(*p*-tolyl)pyridine **20 (** Figure 9 **)**, in which a piperidine replaces the pyrrolidine ring, was shown to increase potency and selectivity over the prototype. Compound **20** displayed high antiproliferative effects in leukemia cell lines such as MV4−11 and Molm-13 (EC_50_ values = 0.36 and 3.4 μM, respectively) as well as in breast cancer cell lines such as MDA-MB-231 and MCF-7 (EC_50_ values = 5.6 and 3.6 μM, respectively). Against non-cancer WI-38 fibroblast cells, **20** exhibited less potency (EC_50_ = 26.6 μM), indicating appreciable cancer-selectivity. Finally, **20** gave accumulation of H3K4me2 in MV4-11 cells, as functional assay to demonstrate its LSD1 inhibition in a cellular context. Crystallographic studies (Niwa et al., 2018) performed on **20** complexed with LSD1/CoREST showed that the piperidine ring penetrated in the LSD1 active site and made interactions with the negatively charged residues Asn540 and Asp555. Moreover, the 4-cyanophenyl group was found deeply in the binding of the substrate pocket and made interactions with Lys661, while the 4-methylphenyl ring bound a hydrophobic cavity.

Several 1,2,3-triazolodithiocarbamates have been reported as novel LSD1 inhibitors (Zheng et al., 2013), with **21** (Figure 9) showing an IC_50_ of 2.1 μM in enzyme assay. In LSD1-overexpressing, low differentiated HGC-27 and MGC-803 human gastric cancer cell lines, **21** exhibited significant reduction of proliferation (IC_50_ values = 1.13 and 0.89 μM, respectively), without toxicity against GES-1 and SGC-7901 non-cancer gastric cell lines (IC_50_ values around 50 μM). In MGC-803 cells, **21** induced up to 44.7% apoptosis (1 μM) after 48 h, and inhibited migration and invasion at sub-toxic doses (0.25–0.02 μM). In MGC-803 xenograft mouse model, **21** at 20 mg/kg reduced the weight of tumor till 68.5%, with no changes in body weight.

Further new LSD1 inhibitors were developed in 2015: among them, the pyrimidine-thiosemicarbazide hybrid compound **22** (Figure 9) proved to be the most potent and selective (IC_50_^LSD1^ = 0.65 µM) (Ma et al., 2015). Furthermore, **22** was found to exert strong and selective cytotoxicity against MGC-803 and HGC-27 gastric cancer cells (IC_50_ = 4–8 μM). Compound **22** also showed inhibitory effects on cell invasion and migration, and tumor suppression and anti-metastatic effects *in vivo* without signs of evident toxicities.

Some indole derivatives (such as **23** and **24**) were also described as reversible LSD1 inhibitors, potent at submicromolar/nanomolar levels. In blood as well as solid cancer cell lines, these compounds exhibited growth arrest at single-digit micromolar concentration (Wang et al., 2020) (Figure 9).

Some [1,2,3]triazolo [4,5-*d*]- and [1,2,3]triazolo [4,5-*a*]pyrimidines, variously substituted with arylthiol, arylamino, thiourea, thiosemicarbazide, and hydrazine moieties, displayed a non-covalent LSD1 inhibition at micromolar/submicromolar level, and inhibited proliferation of a panel of cancer cells at low micromolar doses (Li et al., 2017) (Li Z. et al., 2019) (Wang et al., 2017) (Wang et al., 2019) (Li et al., 2019c).

Starting from the thieno [3,2-*b*]pyrrole-5-carboxamide **25**, selected through an HTS, an optimization process has been undertaken leading to **26**, obtained by elongation of the methoxymethyl substituent at the *meta* position of the **25**’s anilide portion to a 4-piperidinyloxy-4-phenoxymethyl chain. This change gave 18-fold increased potency against LSD1 (Sartori et al., 2017). Further structure-guided optimization furnished some thieno [3,2-*b*]pyrrole-5-carboxamides in which the long chain is moved from the *meta* to the *ortho* position of the anilide ring. Such derivatives showed single-digit nanomolar potency against LSD1 joined to high selectivity over MAOs, and single-digit micromolar (or lower) IC_50_ values against a panel of leukemia cells (MV4-11, THP-1, and NB4) (Vianello et al., 2017). The replacement of the carboxamide linkage with an imidazole (see compound **27** in Figure 9) led to picomolar LSD1 inhibition, together with nanomolar anti-clonogenic activity in THP-1 cells and *in vivo* good efficacy after oral administration in mouse leukemia models (Romussi et al., 2020).

#### 2.2.1 Multi-targeting reversible LSD1 inhibitors

**FIGURE 10:**
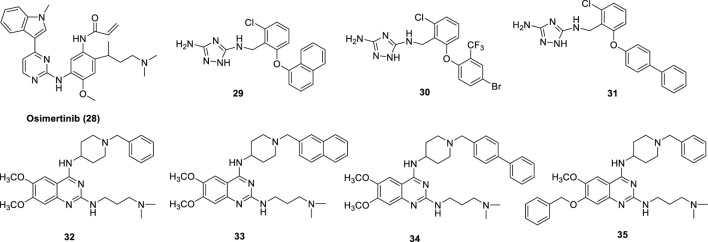
Reversible multi-targeting LSD1 inhibitors.

The EGFR tyrosine kinase inhibitor and antineoplastic agent for the treatment of lung cancer (NSCLC) **Osimertinib** (**28**) (Figure 10) was shown to also inhibit LSD1 (IC_50_ = 3.98 μM). In the treatment of NSCLC, both LSD1 and EGFR are important drug targets. In NCI-H1975 NSCLC cells, Osimertinib reduced cell proliferation with IC_50_ = 3 μM and inhibited migration (Li et al., 2019b). In the same cell line, Osimertinib increased the methylation levels of H3K4me1/2 and abated the level of phospho-EGFR, showing its dual-targeting property in this cellular context. Docking studies revealed that Osimertinib occupies the LSD1 active pocket away from the FAD cofactor. The carbonyl group makes an hydrogen bond with Thr335, while the *N*,*N*-dimethyl group has a ionic interaction with Asp556 (Li et al., 2019b).

A new class of 3,5-diamino-1,2,4-triazoles have been reported as dual LSD1/spermine oxidase (SMOX) inhibitors (**29**-**31**) (Figure 10) (Holshouser et al., 2019). Really, these compounds showed up to 1000-fold difference between the inhibition values of the two targets, and sometimes they are more potent against MAOs than against LSD1.

In 2016, starting from a study on H3K9me1/2 methyltransferases inhibitors (Chang et al., 2010), our group identified **MC3774** (**32**) (Figure 10) (Speranzini et al., 2016) as a compound able to inhibit LSD1 in a unprecedented way. Indeed, five copies of MC3774 stacked in a head-tail orientation at the entrance of the catalytic pocket of LSD1, not allowing the laying of the histone substrate (Speranzini et al., 2016). Furthermore, this complex extensively made interactions with negatively charged residues Glu559, Asp555, Asp557, Asp556, Asp553, and Glu387. MC3774 inhibited at the same time G9a and LSD1 (K_i_ values = 0.68 and 0.44 μM, respectively) and displayed micromolar/submicromolar anticancer activity against leukemia (THP-1 and MV4-11) and solid (breast MDA-MB-231 and rhabdomyosarcoma RD and RH30) cancer cells (Menna et al., 2022). Further chemical manipulation applied on the MC3774 structure furnished derivatives more potent against LSD1 (i.e., **33–35**, K_i_ values = 0.16, 0.15, and 0.11, respectively) and less potent or totally inactive against G9a (K_i_ values = 2.9 (32), 1.2 (33), and 39.4 (34) μM) (Menna et al., 2022). Such derivatives revealed to be more potent than the parent compound against the above leukemia and solid cancer cells, thus suggesting a crucial role for LSD1 in the pathogenesis of these diseases (Figure 10).

#### 2.2.2 Reversible LSD1 inhibitors in clinical trials

**FIGURE 11:**
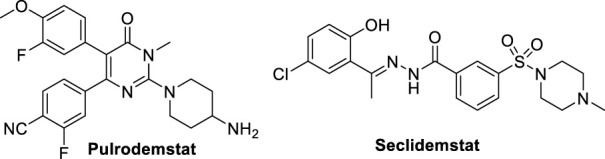
Reversible LSD1 inhibitors in clinical trials.

Despite the huge work performed by researchers for discovery and identification of reversible LSD1 inhibitors (Dai et al., 2021), there are only few of them in clinical trials, i.e. **Pulrodemstat** (**CC-90011**) (Hollebecque et al., 2021) and **Seclidemstat** (**SP-2577**) (Soldi et al., 2020) (Figure 11).

**Pulrodemstat** is the first orally active, reversible LSD1 inhibitor, developed by Celgene (Kanouni et al., 2020). Its structure, identified by a HTS, is similar to those of GSK-690 (**19**) and compound **20**, in which the pyridine is replaced by a pyrimidinone ring, and the cyclic amine-methyloxy group by a 4-aminopiperidine. Pulrodemstat significantly inhibits LSD1 (IC_50_ = 0.30 nM) (Kanouni et al., 2020) and was effective in advanced solid tumors and relapsed/refractory non-Hodgkin’s lymphoma, mainly in patients with neuroendocrine tumors. The pyrimidinone-based LSD1 inhibitor displayed growth arrest against cancer cells *in vitro* as well as in PDX models. Drug combinations of Pulrodemstat with Etoposide and Cisplatin on one side, and with Nivolumab on the other side, have been used against SCLC (NCT03850067) and advanced cancers (NCT04350463), respectively.

**Seclidemstat**, also known as **SP-2577**, is a potent reversible inhibitor of LSD1 (IC_50_ = 13 nM) with no action against MAOs (Sorna et al., 2013). Seclidemstat has received FDA fast track designation for lead drug candidate and has entered phase I clinical trial for the treatment of advanced solid tumors for patients with relapsed/refractory Ewing sarcoma. Reversible inhibitors could ameliorate some possible undesired effects that covalent inhibitors exert on erythropoiesis and establish a novel phenotype of LSD1 inhibition.

In Table 1 the various LSD1 inhibitors, both covalent and reversible, currently in clinical trials for cancer treatment are summarized. The most common causes of treatment failure in cancer patients are metastatic recurrence and therapy resistance.

## 3 Conclusion

In the last 10 years, LSD1 emerged as a potential therapeutic target particularly for the treatment of cancer. In AML and SCLC as well as in other cancer pathologies, elevated levels of LSD1 have been observed. Pharmacological inactivation of LSD1 with small molecule inhibitors showed suppression of cancer cell differentiation, proliferation, invasion, and migration. Numerous LSD1 inhibitors have been reported, with either irreversible or reversible mode of action. Most irreversible LSD1 inhibitors share the TCP structure as the pharmacophore group. They interact with the FAD cofactor to form a covalent bond, giving the irreversible LSD1 inhibition. To date, six TCP-based LSD1 irreversible inhibitors have entered clinical trials (TCP, GSK2879552, Bomedemstat (IMG-7289), Iadademstat (ORY-1001), INCB059872, and Vafidemstat (ORY-2001)). Although their potent and long-lasting effects, irreversible inhibitors have several side effects. For this reason, in the last years, many reversible LSD1 inhibitors have been studied and reported. Until now, only two of them have entered in clinical trials (Pulrodemstat (CC-90011) and Seclidemstat (SP-2577)).

Also, it is important and promising the molecular hybridization approach that combines different drug pharmacophoric moieties, in order to obtain novel dual compounds of two targets, or multitargeting compounds, able to improve potency and selectivity towards cancer cells, ameliorate pharmacokinetics and bioavailability, and possibly reduce adverse effects. Some promising dual-targeting LSD1 inhibitors have been summarized here, with better activity profiles respect to the corresponding single-target inhibitors and their combination.

Due to the important involvements of LSD1 in carcinogenesis, and to the numerous ways with which it interferes with various signaling pathways, targeting lysine demethylases and in particular LSD1 is becoming a promising treatment option for cancer patients.
